# *Paullinia cupana* for control of hot flashes in breast cancer patients: a pilot study

**DOI:** 10.1590/S1679-45082013000400005

**Published:** 2013

**Authors:** Saulo Silva Oliveira, Adriana Braz del Giglio, Tatiana Goberstein Lerner, Rebecca Melo Zanellato, Livia Tiemi, Lucas Reifur, Patrícia Xavier Santi, Auro del Giglio

**Affiliations:** 1 Faculdade de Medicina do ABC, Santo André, SP, Brazil; 2 Faculdade de Medicina do ABC, Santo André, SP, Brazil; Hospital Israelita Albert Einstein, São Paulo, SP, Brazil; Hospital Israelita Albert Einstein, Hospital Israelita Albert Einstein, São Paulo, SP, Brazil

**Keywords:** Breast neoplasms, Hot flashes, Paullinia pinnata, Guarana (Homeopathy)

## Abstract

**Objective:**

To evaluated whether *Paullinia cupana* decrease number and severity of hot flashes in breast cancer survivors.

**Methods:**

This was a prospective phase II pilot study. We studied female breast cancer survivors who had completed the cancer treatment 3 months previously and who were experiencing at least 14 hot flashes per week. At least 9 of the 15 patients were required to have a decrease of at least 50% in hot flash severity score in keeping with the Simon Design. Patients received 50mg of dry extract of *Paullinia cupana* orally twice a day for 6 weeks. We assessed both frequency and severity of hot flashes.

**Results:**

A total of 18 patients started the *Paullinia cupana* treatment, and 15 completed the study. Three patients left the study immediately after starting the treatment because of personal difficulties in participation or noncompliance. Of the 15 patients who completed the study 10 had a decrease of more than 50% in hot flash severity scores. During the 6 weeks of treatment, statistically significant decreases were seen in both numbers of hot flashes (p=0.0009) and severity scores (p<0.0001). *Paullinia cupana* was well tolerated, and there were no instances of discontinuation because of toxicity.

**Conclusions:**

*Paullinia cupana* appears promising for controlling hot flashes. More extensive studies seem warranted.

## RESUMO

**Objetivo:**

Avaliar se a *Paullinia cupana* diminui o número e a gravidade dos fogachos em mulheres após diagnóstico de câncer de mama.

**Métodos:**

Estudo piloto prospectivo fase II realizado com mulheres que sobreviveram ao câncer de mama, que completaram o tratamento pelo menos 3 meses antes e que apresentavam ao menos 14 episódios de fogachos por semana. Utilizando o desenho de Simon para que a primeira etapa fosse considerada positiva, ao menos 9 de 15 mulheres deveriam ter a gravidade dos fogachos diminuída em pelo menos 50%. As pacientes receberam 50mg do extrato seco de Guaraná oralmente 2 vezes por dia por 6 semanas. Foram avaliadas, a gravidade e a frequência dos fogachos.

**Resultados:**

Dezoito pacientes iniciaram o tratamento com *Paullinia cupana* e 15 completaram o estudo. Três pacientes deixaram o estudo imediatamente após iniciarem o tratamento em razão de dificuldade na participação e não adesão. Das 15 pacientes que completaram o estudo, 10 obtiveram diminuição de mais de 50% dos índices de gravidade de fogachos. Durante as 6 semanas de tratamento, diminuições estatisticamente significativas foram observadas tanto no número de fogachos (p=0,0009), quanto nos índices de gravidade (p<0,0001). *Paullinia cupana* foi bem tolerada, e não houve relato de toxicidade como causa de saída do estudo.

**Conclusões:**

*Paullinia cupana* pareceu promissora para o controle de fogachos. Estudos mais extensivos são necessários.

## INTRODUCTION

Hot flashes are episodic sensations of heat, intense sweating and flushing that are often accompanied by palpitations and anxiety^(1,2)^; and they occur in more than 75% of menopausal women^(3)^. Hot flashes are also common among patients with breast cancer^(4)^ and affects up to 70% of those receiving tamoxifen^(4)^.

The mechanisms that lead to the development of hot flashes include the following: (1) dysfunction of the thermoregulatory nucleus with a lowering of central temperature threshold needed to trigger hot flashes; (2) decrease of estrogen levels, which occurs in menopause; and (3) increases in central nervous system levels of norepinephrine and numbers of serotonin receptors^(2)^.

Several attempts to treat hot flashes in breast cancer survivors have been reported. Most of the attempts have used non-hormonal agents such as antidepressants (fluoxetine and venlafaxine) and anticonvulsants (gabapentin)^(2-4)^.

*Paullinia cupana*, also known as *guaraná*, is an Amazonian plant that Brazilian indigenous populations have used for centuries as a tonic because of its antifatigue^(5)^ and thermogenic properties^(6)^.

We report herein our preliminary experience with *Paullinia cupana* to control hot flashes in breast cancer survivors.

## OBJECTIVE

To evaluate the efficacy of *Paullinia cupana* treatment to control hot flashes in breast cancer survivors.

## METHODS

This study was approved by the institutional ethics committee of Faculdade de Medicina do ABC, protocol number 325-2010. The study was from March 2011 to October 2012. We studied women with case history of histologically proven breast cancer who had completed treatment (surgery, adjuvant or neoadjuvant chemotherapy, and radiation therapy) at least 3 months previously. Hormonal therapy of breast cancer with tamoxifen or aromatase inhibitors was permitted. We included only patients who had been experiencing at least of 14 hot flashes per week for at least 1 month. Patients who had received other systemic hormonal agents, such as estrogens, androgens, or progestins for hormonal replacement or any other indication, were excluded. We also excluded patients with decompensated heart failure, arrhythmias or hypertension. Topical vaginal estrogens without expected systemic effects were permitted if used for at least 1 month before entry. A reduction was seen in both the number of hot flashes and their severity. These effects were higher than required to pursue further studies. *Paullinia cupana* seemed to be safe, with no instances of discontinuation of therapy because of side effects. Antidepressants and other systemic nonhormonal treatments for hot flashes, such as vitamin E or soy products, were allowed if routinely used by patients without changes in dosage for at least 1 month before entry into the study.

Patients received 50mg dry extract of *Paullinia cupana* orally twice a day for 6 weeks (Pharmanostra, Rio de Janeiro, Brazil - http://www.pharmanostra.com.br/; batch nº 12030768A). This extract contained 7.97% caffeine and 1.47% tannin. We assessed both frequency and severity of hot flashes.

Medication was discontinued if requested by the patient because of unacceptable side effects. In addition, patients who discontinued for more than 48 hours were removed from the study.

Patients were instructed to complete a daily diary of their hot flashes starting 1 week before the treatment. Each patient was instructed to record each hot flash and classify it as mild, moderate, severe, or very severe. To assist patients in classification, each one received a description of hot flash severity based on previously published reports^(7)^. Patients were evaluated at the beginning of week 2 and weekly thereafter until shortly after week 6; at each visit, the diary of hot flashes was reviewed and the patient was asked about any possible toxicity experienced.

We computed both daily frequency and average severity of hot flashes. Severity scores were 1 for mild, 2 for moderate, 3 for severe, and 4 for very severe^(7)^.

### Statistical methods

This was a prospective, phase II pilot study. The primary objective was to evaluate the efficacy of *Paullinia cupana* in reducing hot flash severity scores. For this endpoint, we considered the percentage of hot flash reduction for each week from the baseline (week 0) and averaged the reductions seen throughout the 6 weeks of the study. A result was considered positive if the average of the reductions from baseline for each of the 6 weeks equaled or exceeded 50%.

We employed the phase II design proposed by Simon^(8)^ to reduce sample size in small pilot trials. We assumed that placebo treatment would reduce the hot flash severity scores by 50% (P0=0.5) in at least 50% of patients. Therefore, postulating that *Paullinia cupana* would cause a 50% reduction of the severity scores in at least 70% of patients (*e. g.*, P1=0.7), we would need 9 or more patients of the 15 to achieve an hot flash severity score reduction of 50%. If 9 or more responses were observed with a type I error of 0.05 and a type II error of 0.20, we could infer a 20% potential superiority (P1-P0=0.20) of *Paullinia cupana* over the expected placebo effect for this one-arm open study.

## RESULTS

In all, 18 patients started *Paullinia cupana* treatment, and 15 completed the 6 weeks of the study. Mean age was 48 (ranged from 36 to 65) years. Of the patients 14 were menopausal, and 1 was pre-menopausal. Three patients left the study shortly after initiate the *Paullinia cupana* treatment because of personal difficulties in participation and noncompliance. Of the 15 patients who completed the treatment, 7 had received adjuvant and 3 neoadjuvant chemotherapy, 6 had received adjuvant radiation therapy, and all patients received adjuvant tamoxifen

**Figure 1:**
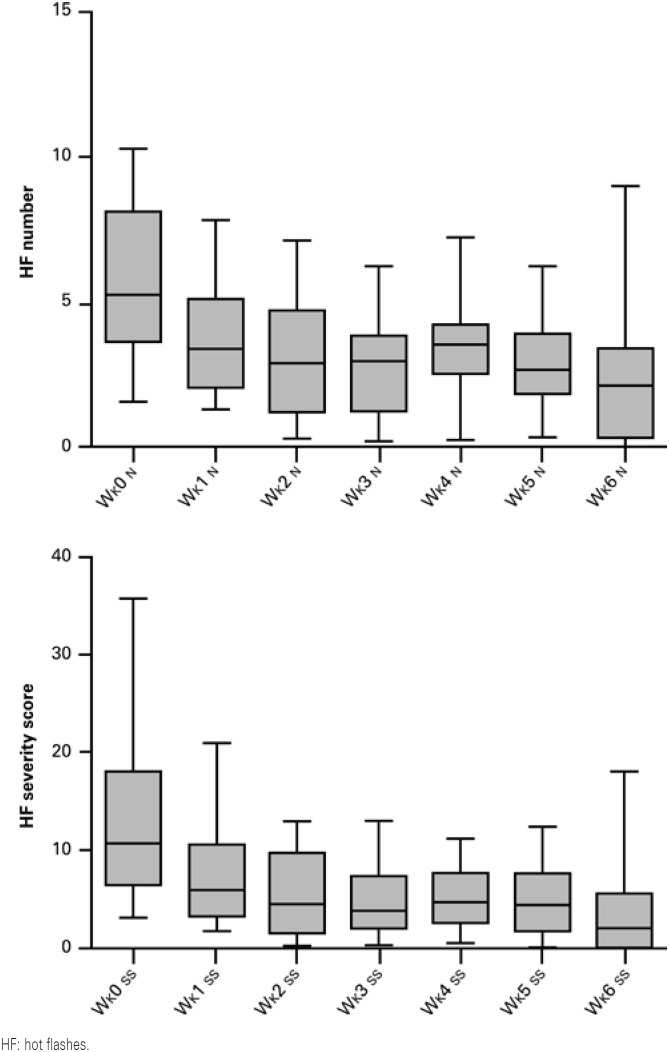
Mean number of hot flashes/day and hot flashes severity scores/day observed during the 6-week treatment with *Paullinia cupana*

Of the 15 patients, 10 had a decrease of more than 50% in hot flash severity score. Throughout the 6 weeks of treatment, we observed statistically significant decreases in both numbers of hot flashes (p=0.0009) and severity scores (p<0.0001) (Figure 1).

Compared with week 0, patients reported worsening of anorexia (1), insomnia (1), nausea (1), fatigue (1), sweating (1), constipation (1), anxiety (1), mood change (1) and headache (1). All signs or symptoms were considered mild and none required patients' discontinuation of the medication.

## DISCUSSION

Hot flashes are a common and distressing symptom that impair quality of life of climacteric women. Breast cancer survivors are of special concern because they often experience hot flashes at an earlier age because of the gonadotoxic effects of anti-neoplastic treatments and the use of agents such as tamoxifen^(3,4)^. Furthermore, breast cancer survivors require non-hormonal options for the control of hot flashes because of the potential harmful effects of hormone replacement in women with hormone-sensitive tumors.

*Paullinia cupana* is an Amazonian plant with tonic properties ascribed to its high caffeine content^(9)^. This plant also has anti-inflammatory effects^(10)^, which might explain the recently described beneficial effects in decreasing the fatigue experienced by breast cancer patients receiving adjuvant chemotherapy^(5)^.

It is unlikely that other concomitant medications such as antidepressants could account for our findings, because patients who were taking these were required to have taken them at least a month before the study and have at least 14 hot flashes a week. We believe that these antidepressants did not have a satisfactory anti-hot flashes activity in those patients.

We observed in this small pilot study a promising effect of *Paullinia cupana* on hot flashes in 10 of 15 patients who had previously experienced at least 14 hot flashes per week.

The underlying mechanism of action observed in effects of *Paullinia cupana* to control hot flashes in this study is unclear. The thermogenic and/or anti-inflammatory properties of *Paullinia cupana*, the potential changes this plant could induce in the central nervous system catecholamines levels might provide perspectives for investigations in the future.

This pilot study employed an experimental design that allowed us to evaluate to use a small sample and no control group. Although this study is not conclusive, the encouraging preliminary results seem to warrant studies using larger samples of patients and a placebo control arm.

## CONCLUSION

A reduction was seen in both the number of hot flashes and their severity. These effects were higher than required to pursue further studies. *Paullinia cupana* seemed to be safe, with no instances of discontinuation of therapy because of side effects. This promising agent should be studied further.
